# Supplementary figures and images for: Identification and Expression Analysis of Dsx and Its Positive Transcriptional Regulation of IAG in Black Tiger Shrimp (Penaeus monodon)
Source: Int J Mol Sci. 2022 Oct 21;23(20):12701. doi: 10.3390/ijms232012701 (PMC9604489; doi:10.3390/ijms232012701)

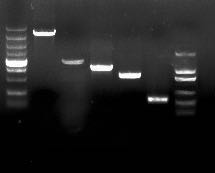

Supplement: Supplementary file 1 [file ijms-23-12701-s001.zip › Figure S1 Amplification of different segments of IAG promoter.jpg]
